# Cancer-Associated Exosomal CBFB Facilitates the Aggressive Phenotype, Evasion of Oxidative Stress, and Preferential Predisposition to Bone Prometastatic Factor of Breast Cancer Progression

**DOI:** 10.1155/2022/8446629

**Published:** 2022-07-19

**Authors:** Chia-Hung Hsu, Hon-Ping Ma, Jiann Ruey Ong, Ming-Shou Hsieh, Vijesh Kumar Yadav, Chi-Tai Yeh, Tsu-Yi Chao, Wei-Hwa Lee, Wen-Chien Huang, Kuang-Tai Kuo, Iat-Hang Fong, Chih-Cheng Lin, Chih-Ming Su

**Affiliations:** ^1^Department of Emergency Medicine, Shuang Ho Hospital, Taipei Medical University, New Taipei City, Taiwan; ^2^Graduate Institute of Injury Prevention and Control, College of Public Health, Taipei Medical University, Taipei City, Taiwan; ^3^Department of Emergency Medicine, School of Medicine, Taipei Medical University, Taipei, Taiwan; ^4^Department of Medical Research & Education, Taipei Medical University-Shuang Ho Hospital, New Taipei City 235, Taiwan; ^5^Department of Biotechnology and Pharmaceutical, Yuanpei University of Medical Technology, Hsinchu City 30015, Taiwan; ^6^Graduate Institute of Clinical Medicine, College of Medicine, Taipei Medical University, Taipei 110, Taiwan; ^7^Department of Pathology, Taipei Medical University-Shuang Ho Hospital, New Taipei City, Taiwan; ^8^Department of Medicine, MacKay Medical College, Taipei 110, Taiwan; ^9^Division of Thoracic Surgery, Department of Surgery, MacKay Memorial Hospital, Taipei 110, Taiwan; ^10^Division of General Surgery, Department of Surgery, School of Medicine, College of Medicine, Taipei Medical University, Taipei City, Taiwan; ^11^Division of General Surgery, Department of Surgery, Department of Surgery, Taipei Medical University Shuang Ho Hospital, New Taipei City, Taiwan

## Abstract

**Background:**

Despite therapeutic advancements, metastasis remains a major cause in breast cancer-specific mortality. Breast cancer cells are susceptible to oxidative damage and exhibit high levels of oxidative stress, including protein damage, DNA damage, and lipid peroxidation. Some breast cancer risk factors may change the level of endogenous oxidative stress. Circulating exosomes play critical roles in tumorigenesis, distant metastasis, and poor prognosis in patients with breast cancer.

**Methods:**

We used an online database to analyze the expression and prognostic value of core binding factor subunit *β* (CBFB) and oxidative stress–related targets in patients with breast cancer. Serum from healthy controls and patients with primary breast cancer or bone metastatic breast cancer in the bone was collected. Exosomes were isolated from the sera or cell culture media. We used an MDA-MB-436-innoculated tumor xenograft mouse model for silencing CBFB.

**Results:**

Circulating exosomes from patients with breast cancer metastasis to the bone were rich in CBFB. The human mammary fibroblast cells HMF3A and fibroblasts derived from patient samples cocultured with exosomes had increased *α*-SMA and vimentin expression and IL-6 and OPN secretion. Similarly, nonmetastatic breast cancer cells cocultured with exosomes exhibited increased levels of certain markers, including vimentin, snail1, CXCR4, and Runx2, and the exosomes had high CBFB expression. Silencing CBFB in metastatic MDA-MB-436 and MDA-MB-157 cells resulted in suppressed migration and invasion and downregulation of vimentin, CXCR4, snail1, Runx2, CD44, and OPN. Conversely, CBFB overexpression resulted in upregulation of Runx2, vimentin, snail1, CD44, and OPN in nonmetastatic T47D and MCF12A cells. The CBFB-rich exosomes derived from MDA-MB-436 cells induced enhanced metastatic phenotypes in the low-metastatic T47D and MCF12A cell lines.

**Conclusion:**

Our results revealed that CBFB may promote bone metastasis in patients with breast cancer. Of therapeutic relevance, targeting CBFB resulted in decreased tumor burden and bone metastasis, downregulation of bone metastasis markers, and impaired regulation of oxidative stress–related proteins NAE1 and NOS1.

## 1. Introduction

Breast cancer is a common cancer in women, accounting for approximately 30% of all new cancer diagnoses among women in the United States in 2016 [1, 2]. Despite therapeutic advancements, treatment options for patients with distant metastases remain limited, and the efficacy is dismal. This is compounded by the lack of reliable prognostic and therapeutic biomarkers, necessitating the identification of novel therapeutic targets and markers, especially for patients with advanced stage disease.

Extracellular vesicles or exosomes (30–150 nm) are secreted by various cell types and transport a wide range of cargoes, including nucleic acids (DNA, mRNAs, and noncoding RNAs), proteins (cytoskeletal proteins, cytokines, and heat shock proteins), and enzymes (e.g., GAPDH and ATPase) [3, 4]. Proteins such as growth factors and interleukins are transported by tumor-derived exosomes, and these regulate various biological processes [5]. Intracellular communication is accomplished by exosomal exchanges in various cell types. Exosomes are involved in many aspects of tumorigenesis, including immune suppression, angiogenesis, cell migration, and invasion [6–8]. Because they facilitate intercellular transfer of signaling molecules between the donor and recipient cells within the tumor microenvironment (TME) or to distant sites [9], tumor-derived exosomes represent a powerful tool by which cancer cells promote malignant transformation of normal cells. Annexin II-enriched exosomes promote metastasis through enhanced angiogenesis and create a more favorable TME in breast cancer [10].

Notably, the core-binding factor *β* subunit (CBFB) plays various critical roles in breast cancer. CBFB, a transcription factor that interacts with RUNX family transcription factors, activates its target genes and plays key roles in normal development and diseases in humans. Studies using xenograft mouse models have revealed that its knockdown suppressed ovarian and prostate tumor growth [11]. CBFB upregulation enhances the resistance of acute myeloid leukemia cells to a novel antileukemic RUNX inhibition therapy [12]. By contrast, in breast cancer, CBFB overexpression suppresses the NOTCH oncogenic signaling pathway and migration ability of the breast cancer cells [13, 14]. However, another study reported that CBFB upregulation is required to maintain the invasive ability of breast cancer cells [15]. Given these contradictory roles of CBFB in breast cancer, we investigated the role of CBFB in breast cancer development and progression. Notably, we provide evidence that CBFB is implicated in breast cancer metastasis to the bones and that this is associated with the exosomal enrichment of CBFB in the serum of patients with breast cancer.

Reactive oxygen species (ROS) have multiple effects at the initial stage of carcinogenesis, including activation of cancerous substances, DNA damage, and DNA damage response. Compared with normal cells, cancer cells have higher ROS levels as well as increased antioxidant defense to balance the oxidation state; nevertheless, high ROS levels can prevent carcinogenesis through various mechanisms. ROS are produced by various metabolic pathways, including aerobic metabolism in the mitochondrial respiratory chain. ROS affects different signaling pathways, including growth factors and mitotic pathways, and controls many cellular processes, including uncontrolled cell proliferation. The increase in oxidative stress caused by ROS can reduce the body's antioxidant defense against angiogenesis and metastasis of cancer cells, which is the main process of cancer development. ROS-mediated oxidative stress plays a role in the pathogenesis of breast cancer through genetic and epigenetic modifications, leading to uncontrolled cell proliferation. The role of oxidative stress in the induction and progression of breast cancer can be attributed to impaired balance between prooxidants and antioxidants.

The TME facilitates breast cancer progression, especially in terms of distant metastasis. One of the most important players in the TME is the cancer-activated/associated fibroblasts (CAFs). CAFs secrete cytokines such as platelet-derived growth factors and transforming growth factor beta (TGF-*β*) to recruit other fibroblasts and immune cells to promote angiogenesis and tumor growth [16]. Furthermore, exosomes secreted within the TME facilitate cellular communications between stromal cells and cancer cells and subsequently drive distant metastasis [1, 2]. Notably, coculturing breast cancer–derived exosomes with normal fibroblast induced acquisition of CAF phenotypes by normal fibroblasts [17]. However, the mechanism by which exosomes induce CAF phenotypes and associated disease progression remains poorly understood.

Considering that tumor-derived exosomes may promote breast cancer progression, we investigated their roles in promoting bone metastasis and the contribution of altered exosomal CBFB levels to these processes. Moreover, we examined the effects of tumor-derived CBFB-rich exosomes on the acquisition of CAF-like phenotypes and the associated metastatic and oxidative stress potential of breast cancer cells. Our results may guide the future development of therapeutics against metastatic breast cancer.

## 2. Methods

### 2.1. Clinical Samples, Cell Lines, and Cell Culture

Clinical samples were obtained from patients with breast cancer. The procurement of the samples adhered strictly to the protocol approved by the Joint Institutional Review Board of Shuang Ho Hospital (N201603028). Nontumor (NT) and tumor (T) tissues were collected from the Shuang Ho Hospital breast cancer tissue archives. Nonmetastatic human breast cancer cell lines MCF12A and T47D, metastatic triple-negative breast cancer (TNBC) cell lines MDA-MB-157 and MDA-MB-436, and the human mammary fibroblast cell line HMF3A were all purchased from the American Type Culture Collection (Manassas, VA, USA). All cell lines were cultured in Dulbecco's Modified Eagle's Medium (DMEM) (DML10-1000ML, Caisson Labs, Smithfield, UT, USA) supplemented with 10% fetal bovine serum (FBS), 100 U/mL penicillin, and 100 *μ*g/mL streptomycin in 5% CO_2_ humidified atmosphere at 37°C. For maintenance, all cell lines were subcultured every 48–72 h.

### 2.2. CBFB Knockdown and Overexpression

Lentivirus containing CBFB short hairpin (sh) RNA was purchased from Thermo Fisher Scientific (USA) and prepared strictly as per the manufacturer's instructions. Two clones of shRNA were used to effectively silence CBFB expression: A6 (shRNA1, clone ID: V2LHS-89195) and B10 (shRNA2, V3LHS-639151). shRNA lentivirus infection and construction were conducted according to standardized practice guidelines in our certified BSL-2 laboratory in the Integrated Laboratories for Translational Medicine, TSGH. Lipofectamine 3000 (L3000008, Thermo Fisher Scientific, Waltham, MA, USA) was used for siRNA transfection following the manufacturer's protocol. CBFB overexpression was conducted using the Ultimate ORF Clones (Cat No. IOH46204, CBFB-OE, Thermo Fisher Scientific). The full-length CBFB fragments were subcloned to pcDNATM3.1 vector, using the Gateway Invitrogen recombination cloning system according to the protocols provided by the manufacturer. CBFB knockdown and overexpression were confirmed using an anti-CBFB antibody (Cat. No. PA1-317, Thermo Fisher Scientific), as demonstrated by Western blot assays.

### 2.3. Exosome Isolation

Sera from healthy controls, patients with primary breast cancer, and metastatic breast cancer to the bones were collected. For isolation from cell lines, after culturing was conducted wild-type (negative control), shCBFB-transfected, or CBFB-OE-transfected breast cancer cell lines in serum-free medium for 48 h, the culture media were collected. Exosomes were isolated from collected serum and culture media as previously described [18]. Briefly, after repeated centrifugation and ultracentrifugation (500 × g for 10 min, 1200 × g for 20 min, and 10,000 × g for 30 min), filtration with 0.22 *μ*m pore syringes, and a spin at 100,000 × g for 60 min, the cell pellet was collected, washed in 1× PBS three times, and ultracentrifuged at 100,000 × g for 60 min; subsequently, the exosomes were harvested from the supernatant. The harvested exosomes were used for further analysis. Western blot analysis with anti-CD63 (Cat No. H5C6, Novus Biologicals, CO, USA) and anti-CD9 (Cat No. 5G6, Novus Biologicals) antibodies was used to confirm the presence of exosomes.

### 2.4. Cytokine Estimation

The concentration of IL-6 and OPN was measured using sandwich enzyme immunoassay kits (Cat. No. LS-F9982 and LS-F171, respectively; LSBio, Seattle, WA, USA). The experiments were performed according to the manufacturer's instructions, and the concentration of IL-6 and OPN was determined using interpolation from a standard curve.

### 2.5. Real-Time Polymerase Chain Reaction Analyses

For real-time polymerase chain reaction analyses, tumor cells or fibroblasts (2 × 10^4^) coincubated with exosomes (10 *μ*g protein) for 48 h were harvested and lysed using RLT buffer (QIAGEN, Hilden, Germany). Total RNA was isolated using a RNeasy Kit (QIAGEN). cDNA was obtained using TaqMan in the StepOnePlus system (Applied BioSystems, MA, USA). All experimental cycle threshold (Ct) values were normalized against the Ct value of the internal control GAPDH. Relative abundance was determined using 2-*ΔΔ*Ct and expressed as fold changes. The primers used in this study are listed in Supplementary Table S1.

### 2.6. Western Blot Analysis

Total protein lysates were obtained and isolated from cancer cell lines using the radioimmunoprecipitation (RIPA) lysis buffer supplemented with protease inhibitor (1x, Cat# 78430, Thermo Fisher Scientific) and phosphatase inhibitor (0.5x, Pierce Phosphatase Inhibitor Mini Tablets, Thermo Fisher Scientific). A bicinchoninic acid assay Protein Assay Kit (Thermo Fisher Scientific) was used to determine the final protein concentration. Total protein samples were separated by standard sodium dodecyl sulfate–polyacrylamide gel electrophoresis using the Protean III system (Bio-Rad, CA, USA) and transferred onto a polyvinylidene fluoride membrane using the Trans-Blot Turbo Transfer System (Bio-Rad). The primary antibodies against Snail (Cat. No. 3895, 1 : 1000), vimentin (Cat No. 5741, 1 : 1000), CD44 (Cat No. 5640, 1 : 1000), CXCR4 (Cat No. 97680, 1 : 1000), and Runx2 (Cat. No. 12556, 1 : 500) purchased from Cell Signaling Technology, (MA, USA) and anti-OPN (Cat No. ab69498, 1 : 500) purchased from Abcam (Cambridge, UK) were used in this study. These are listed in Supplementary Table S2.

### 2.7. Coimmunoprecipitation (CO-IP)

Immunoprecipitation (IP) is an experimental technique based on antibodies, which can isolate target proteins from cell. Here, coimmunoprecipitation was used to detect CBFB–Runx2 interaction in vitro. Nondenaturing lysis buffer (20 mM Tris HCl pH 8, 137 mM NaCl, and 1% Nonidet P-40, 2 mM EDTA) store in 4°C and immediately before use add protease inhibitors. Place the cell culture dish on ice and wash the cells with ice-cold PBS. Then, add ice-cold lysis buffer. After that, cells are completely lysed under nondenaturing conditions, and proteins that bound together are kept. The workflow is divided into three parts. First, mix the specific antibody with the sample to capture the target protein. Add the Protein A or G affinity chromatography colloid (or magnetic beads) to form an “antigen-antibody-colloid” complex. Irrelevant, nonbinding proteins, antigens, and any proteins that are bound are eluted by series of washes. Then, the bound proteins which eluted are analyzed by SDS-PAGE/immunoblotting.

### 2.8. Wound Healing and Matrigel Cell Invasion Assays

For the wound healing migration assay, MDA-MB-436 and T47D cells were seeded in six-well plates and cultured until 100% confluence. Next, sterile 200 *μ*L micropipette tips were used to create same-size scratch wounds along the median axis of the monolayer of adherent cells. The human breast cancer cells migration ability demonstrated by wound gap closure was monitored over time, and photographs were taken immediately after scratch wounds were created and at indicated time points. Invasion assays were performed using the 24-well plate Transwell system (8 *μ*m pore size) polycarbonate filter (#ECM508, Millipore, MA, USA). The lower chamber contained 400 *μ*L DMEM supplemented with 10% FBS and 10 ng/mL rhEGF. The upper chamber was precoated with solubilized Matrigel (1 mg/mL, Corning, NY, USA) and seeded with 1 × 10^4^ breast cancer cells (0.1% FBS media) preincubated with or without exosomes for 48 h. After 24 h incubation, the media were discarded; uninvaded cells on the upper side of the inserts were carefully removed with sterile cotton buds, whereas the invaded cells on the lower side of the filter membrane were fixed with 4% formaldehyde for 1 h and stained with 0.1% (*w*/*v*) crystal violet solution for 20 min. The cells were imaged and evaluated under a microscope.

### 2.9. Tumorsphere Formation Assay

The in vitro spheroid formation assay is a common assay used to measure the self-renewal and multipotent nature of the cancer stem cell subpopulations within a tumor or cancer cell line. For tumorsphere formation, we seeded MDA-MB-436 and T47D human breast cancer cell lines in six-well ultralow attachment plates (Corning Inc., Corning, NY, USA) containing DMEM/F12 medium supplemented with B27 (Invitrogen, Carlsbad, CA, USA), 20 ng/mL EGF (Millipore, Bedford, MA, USA), and 20 ng/mL bFGF (Invitrogen, Carlsbad, CA, USA).

### 2.10. Flow Cytometry

PE-VIM/*α*-SMA staining for detection of cell death was performed using the BD FACSCanto II flow cytometry system (BD Biosciences, San Jose, CA, USA) following the manufacturer's instructions. The cells were incubated with PE-labeled vimentin (VIM) and alpha-smooth muscle actin (*α*-SMA) at room temperature for 30 min and then analyzed using flow cytometry. The mitochondrial transmembrane potential (D*Ψ*m) was evaluated using the cationic dye JC-1 (Mitochondrial Membrane Potential Assay Kit, #ab113850, Abcam) following the manufacturer's instructions (BD Pharmingen); 1 × 10^6^ HMF3A cells were incubated with 10 *μ*g/mL JC-1 at 37°C in the dark for 15 min and then analyzed using flow cytometry. All samples were assayed three times in triplicate.

### 2.11. *In Vivo* Analysis of the Effect of CBFB in Tumor Xenograft Mouse Models

The animal experiments were performed strictly according to the protocols approved by Taipei Medical University (affidavit of approval of animal use protocol # LAC-2017-0129). In total, 2 × 10^6^ MDA-MB-436 (control wild-type and sh-CBFB) was injected subcutaneously into the right flank of NOD/SCID mice (6-week-old females, *N* = 5 per group) purchased from BioLASCO (Taipei, Taiwan). Tumor growth was monitored on a weekly basis, and tumor volume was measured using the standard caliper method and calculated using the formula: tumor volume (*V*) = (*W*2 × *L*)/2, where *W* is the shortest diameter and *L* is the longest diameter of the tumor. The survival of mice from the control wild-type and sh-CBFB groups was monitored and analyzed using the Kaplan–Meier survival curve. At the end of the in vivo study, animals were humanely killed by cervical dislocation, and tumor samples were harvested for further analyses.

### 2.12. Hematoxylin and Eosin Staining

Tissues collected from the animals were fixed in 10% (vol/vol) formalin for 24 h and embedded in paraffin. Bones were decalcified prior to paraffin embedding. Subsequently, 4 *μ*m thick tissue sections were dewaxed by incubating the sections with xylene for 2 min twice and rehydrating them with 100% ethanol twice for 2 min, 95% ethanol for 2 min, 75% ethanol for 2 min, and ddH_2_O for 2 min. The tissue sections were stained with hematoxylin for 2 min, washed with tap water for 10 min, counterstained with eosin for 30 s, and then dehydrated with 75% ethanol for 30 s twice, 95% ethanol for 30 s, 100% ethanol, and xylene for 30 s. After being mounted with mounting medium, the stained tissue sections were examined under a light microscope, and the tumor areas were digitally photographed. The tumor areas were calculated using ImageJ version 1.5 software (Wayne Rasband National Institutes of Health, Bethesda, MD, USA).

### 2.13. Statistical Analysis

All the experiments were performed at least three times in triplicate, and the results are expressed as mean ± standard deviation. All statistical analyses, including the overall survival analysis using the Kaplan–Meier plots, were performed with GraphPad Prism for Windows version 7.00 (GraphPad Software, San Diego, CA, USA). Quantitative analyses of migration and invasion assay data were performed using ImageJ version 1.5 (Wayne Rasband National Institutes of Health). Student's *t*-test was used to compare the data between the control and experimental groups. A one-way analysis of variance was used to compare ≥3 groups. *p* < 0.05 was set as statistically significant, with ^∗^*p* < 0.05, ^∗∗^*p* < 0.01, and ^∗∗∗^*p* < 0.001.

## 3. Results

### 3.1. CBFB Expression Is Upregulated in TNBC Cells and Correlates with Poor Prognosis

**Figure 1:**
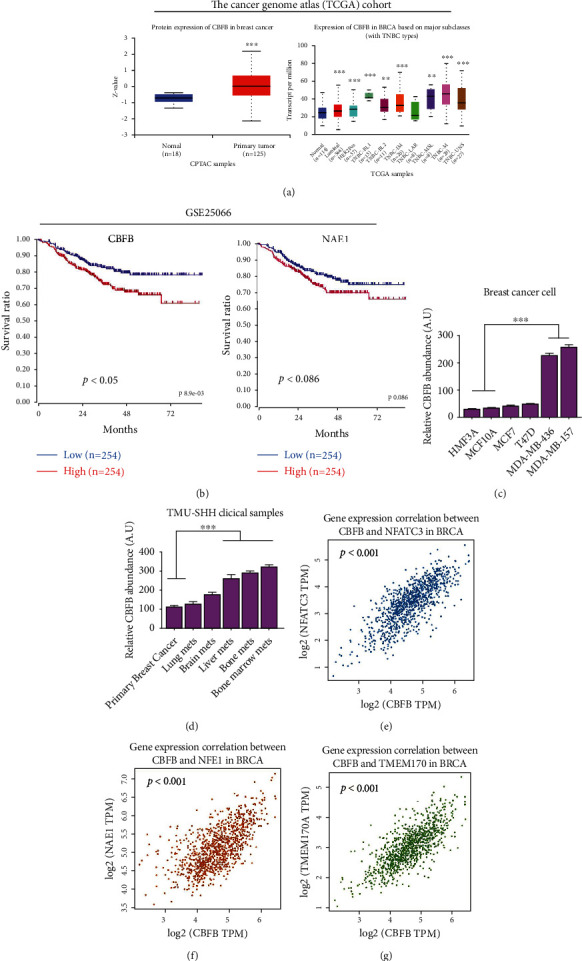
CBFB expression is upregulated in triple-negative breast cancer (TNBC) cells and correlates with poor prognosis. (a) The Cancer Genome Atlas (TCGA) cohort exhibits abnormal CBFB expression in breast cancer and triple-negative breast cancer tissues. Increased CBFB expression is associated with metastasis and poor prognosis in patients with breast cancer. (b) A Kaplan–Meier metastasis-free survival curve derived from GSE25066 breast cancer cohort (*N* = 508) reveals a shorter metastasis-free survival time for patients with a higher level of CBFB and NAE1. (c) Comparative qPCR analysis of CBFB expression between immortalized human mammary fibroblast (HMF3A), normal-like breast (MCF10A), low-metastatic-potential (MCF12A and T74D), and metastatic breast cancer (MDA-MB-436 and MDA-MB-157) cell lines. We observed the CBFB mRNA expression level in HMF3A, MCF10A, MCF12A, and T74D did not differ significantly. CBFB mRNA level was significantly higher in both metastatic cell lines, MDA-MB-436 and MDA-MB-157, than in normal-like breast cell line (MCF10A) and low-metastatic-potential MCF12A and T47D counterparts. (d) Different CBFB mRNA expression between nonmetastasis versus bone metastasis samples. (e–g) The expression of CBFB and oxidative stress–related targets NFATC3, NAE1, and TMEM170A. Patients with bone metastasis exhibited the highest CBFB among all samples (graph generated from http://gepia2.cancer-pku.cn/#survival). Each group consists of 10 samples. NS: not significant; ^∗∗^*p* < 0.01, ^∗∗∗^*p* < 0.001.

The Cancer Genome Atlas (TCGA) cohort exhibited abnormal CBFB expression in breast cancer and TNBC tissues. Increased CBFB expression was associated with metastasis and poor prognosis in patients with breast cancer (Figure 1(a)). In analyzing the GSE25066 cohort, patients with high CBFB expression and NAE1 had poor survival rates (Figure 1(b)). Among them, CBFB expression was statistically significant (*p* < 0.05), and NAE1 does not differ significantly (*p* = 0.086). The cohort is a microarray-based gene expression test from pretreatment breast cancer biopsies (310 patients) to predict favorable outcome based on estrogen receptor (ER) status, pathologic response to chemotherapy, 3-year disease outcomes, and sensitivity to endocrine therapy. Moreover, compared with the very low CBFB expression in HMF3A cells and the nontumorigenic breast epithelial cell line MCF10A, CBFB expression was mildly elevated in the MCF7 and T74D breast cancer cell lines with low metastatic potential and markedly upregulated in the highly metastatic MDA-MB-436 and MDA-MB-157 cells (CBFB: MDA-MB-436, MDA-MB-157>>MCF7, T47D>MCF10A, HMF3A) (Figure 1(c)). Similarly, expression data from the cancer cell line encyclopedia indicated that CBFB expression in primary breast cancer cell lines was significantly lower than that in the metastatic breast cancer cell lines (Supplementary Fig. S1). CBFB expression was the highest in breast cancer cell lines derived from brain and skin metastatic sites. Thus, to explore the probable role of CBFB in breast cancer metastasis, we assessed CBFB expression in primary and metastatic (the lung, brain, and bone) breast cancer tissues. No significant difference was observed in the CBFB level between breast cancer with metastasis to the lungs and primary breast cancer tissues; CBFB expression was significantly upregulated in breast cancer with metastasis to the bone (1.64-fold, *p* < 0.01) and brain (2.41-fold, *p* < 0.001). These data suggest the tissue specificity of CBFB expression in patients with metastatic breast cancer (Figure 1(d)). Finally, we analyzed the relationship between the expression of CBFB and oxidative stress–related targets (NFATC3 and NAE1). The results showed that CBFB was statistically significant with both (*p* < 0.001). A recent study indicated that TMEM170A is a new regulator of endoplasmic reticulum and nuclear membrane morphogenesis, whereas CBFB expression of TMEM170A also has a positive correlation trend and was significant (*p* < 0.001) were shown in Figures 1(e)–1(g).

### 3.2. Serum Exosomes Rich in CBFB Promote the Metastatic Potential of Breast Cancer Cells

**Figure 2:**
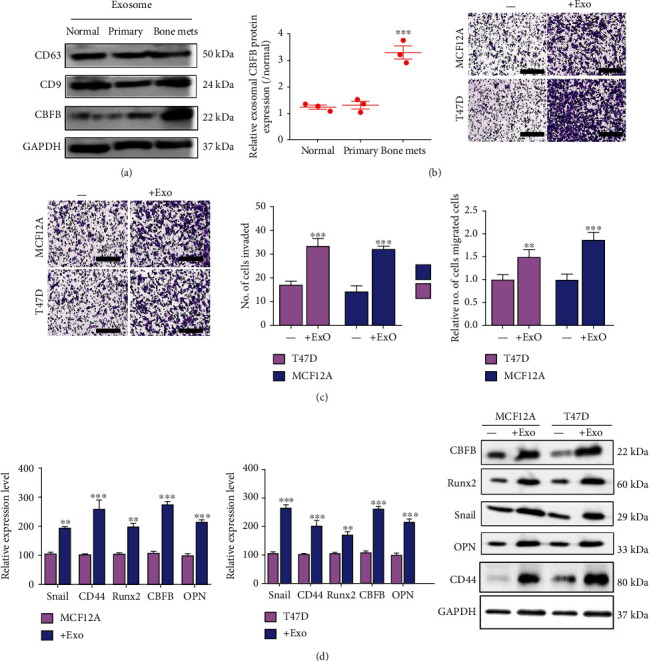
CBFB-rich exosomes promoted metastatic potential in vitro. (a) Exosomes from bone metastatic patients exhibited significantly higher CBFB levels than those from primary and control patient counterparts. CD63 and CD9 was assessed to confirm the existence of exosomes. Coculture of serum exosomes from bone metastatic patients significantly increased the migration (b) and invasion (c) abilities of low-metastatic-potential breast cancer cell lines MCF12A and T47D. Representative images of invasion are shown. Scale bars: 200 *μ*m. (d) Analytical qPCR and Western blotting results demonstrated that exosome-treated MCF12A and T47D cells expressed a markedly higher level of Snail, CD44, OPN, CBFB, and Runx2 compared with their controls. NS: not significant; ^∗∗^*p* < 0.01, ^∗∗∗^*p* < 0.001.

Serum exosomes have increasingly been explored for their varied roles in the prognoses of different diseases, including breast cancer [9, 19]. We observed that the exosomes from patients with bone metastatic breast cancer had markedly higher CBFB expression than those from healthy controls and patients with primary breast cancer (Figure 2(a)). Notably, when breast cancer cell lines with low metastatic potential—T47D and MCF12A—were incubated with exosomes derived from patients with bone metastatic breast cancer, a significant increase in migration (T47D: 1.46-fold, *p* < 0.01; MCF12A: 1.9-fold, *p* < 0.001; Figure 2(b)) and invasion (T47D: ~2-fold, *p* < 0.001; MCF12A: 2.6-fold, *p* < 0.001) abilities was observed (Figure 2(c)). This was associated with the upregulation of CBFB, Runx2, epithelial-to-mesenchymal transition (EMT) markers Snail and CD44, and the bone metastasis marker osteopontin (OPN) at both mRNA and protein levels (Figure 2(d)).

### 3.3. CBFB-Rich Exosomes Promote the Acquisition of a CAF-Like Phenotype

**Figure 3:**
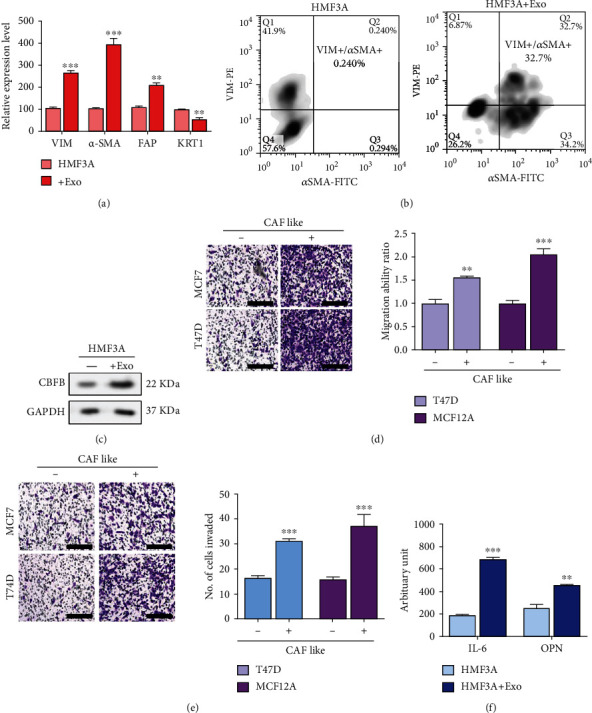
Exosomes rich in CBFB promoted the generation of CAF-like phenotype. (a) HMF3A cells treated with CBFB-rich exosome derived from bone metastatic patients with breast cancer (exosomes) exhibited significant increases in CAF markers, including vimentin (Vim), FAP, and *α*-SAM, and decreases in cytokeratin (KRT1) in immortalized human mammary fibroblast cell line HMF3A. (b) The CAF-like (VIM+/*α*SMA+) cell percentages increased following the exosomes treatment. (c) The Western blot results demonstrated CBFB upregulation in the HMF3A cells treated with exosomes. HMF3A-exosomes cocultured with low-metastatic-potential breast cancer cell lines exhibited enhanced metastatic potential. Both low-metastatic-potential breast cancer cell lines, MCF12A and T47D, exhibited enhanced migration (d) and invasion (e) abilities compared with their control counterparts. Representative images of invasion are shown. Scale bars: 200 *μ*m. (f) ELISA analyses of exosome-induced CAFs. CAFs generated from exosome coculture demonstrated an increased secretion of IL-6 and OPN into the culture medium. ^∗∗^*p* < 0.01, ^∗∗∗^*p* < 0.001.

The TME plays a central role in cancer progression. In particular, CAFs have been implicated as key players in the TME that promote distant metastasis and drug resistance of breast cancer cells [16]. In this study, we demonstrated that coculture of HMF3A with exosomes resulted in a significant increase in the expression levels of CAF markers vimentin (2.78-fold, *p* < 0.001), *α*-SMA (3.91-fold, *p* < 0.001), and fibroblast activation protein alpha (FAP) (2.1-fold, *p* < 0.01), with concurrent downregulation of cytokeratin 1 (KRT1) (Figure 3(a)). To assess the proportion of CAF-like cells, we performed flow cytometry probing of vimentin (VIM-PE) and *α*-SMA (*α*SMA-FITC). Remarkably, HMF3A cells cocultured with exosomes displayed a significant increase in the VIM+/*α*-SAM+ (CAF-like) cell proportions (136.25-fold, *p* < 0.01) (Figure 3(b)). Concurrently, Western blotting results revealed that the exosome-cocultured HMF3A had higher CBFB expression levels than did the noncocultured HMF3A cells (Figure 3(c)). These findings suggest that the exosomes are rich in CBFB and that the acquired CAF-like phenotype of the HMF3A cells is attributable to enhanced CBFB levels. Subsequently, we cocultured the CAF-like cells (generated from HMF3A cells incubated with exosomes), with T47D or MCF12A cells. We observed significantly enhanced metastatic potential in both cell lines as reflected by their increased migration (T47D: 1.71-fold, *p* < 0.01; MCF12A: 2.13-fold, *p* < 0.001; Figure 3(d)) and invasion (T47D: 2.06-fold, *p* < 0.001; MCF12A: 1.87-fold, *p* < 0.001; Figure 3(e)) abilities. Furthermore, compared with the singly cultured HMF3A cells, HMF3A cells cocultured with exosomes displayed a markedly increased secretion of IL-6 (3.68-fold, *p* < 0.001) and OPN (2.04-fold, *p* < 0.01) into the culture medium (Figure 3(f)).

### 3.4. CBFB Levels Are Associated with Oxidative Stress and the Metastatic Potential of Breast Cancer Cells In Vitro

**Figure 4:**
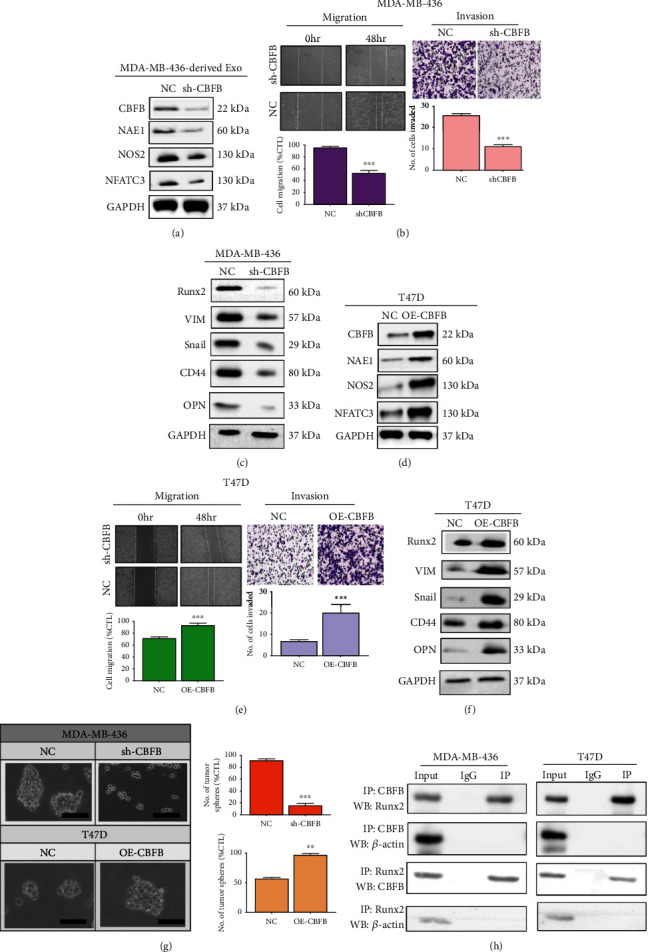
CBFB expression was associated with oxidative stress and metastatic potential of breast cancer cells. (a) Western blot images indicating the effect of CBFB-knockdown on the expression of CBFB, NAE1, NFATC3, NOS2, and PARP1 in highly metastatic MDA-MB-436 cells. (b) CBFB-knockdown MDA-MB-436 cells (sh) exhibited significantly reduced migration (left panels) and invasion (right panels) abilities. The insert represents the Western blots indicating CBFB was silenced compared with negative control (NC). (c) Western blots demonstrating the significantly reduced expression of Snail, vimentin (Vim), OPN, CD44, and Runx2 in CBFB-knockdown MDA-MB-436 cells compared with their parental counterparts (NC). (d) Western blot images indicating the effect of CBFB-knockdown on the expression of CBFB, NAE1, NFATC3, and NOS2 in nonmetastatic T47D cells overexpressed with CBFB. (e) CBFG-overexpressing (OE) T47D cells exhibited enhanced migration (left panels) and invasion (right panels) abilities compared with their parental counterparts (NC). Representative images of invasion are shown. Scale bars: 200 *μ*m. (f) Western blots comparing parental and CBFB-overexpressing T47D cells. Metastasis-associated markers Snail, CD44, Vim, OPN, and Runx2 were all elevated in the OE cells. (g) Tumorsphere formation from MDA-MB-436 and T47D. (h) CO-IP experiment checks the interaction between factors affected after altering CBFB expression, ^∗∗∗^*p* < 0.001. *β*-Actin and GAPDH are the loading control.

We used the gain-of-function and loss-of-function approaches to investigate the role of CBFB in the evasion of cellular stress and apoptosis and in breast cancer cell metastasis. First, we observed that silencing CBFB suppressed CBFB, NFATC3, and NOS2 expressions (Figure 4(a)). Moreover, CBFB knockdown in metastatic MDA-MB-436 cells resulted in significantly reduced migration and invasion ability (Figure 4(b)) and markedly downregulated Runx2, vimentin, Snail, CD44, and OPN (Figure 4(c)). Conversely, compared with their wild-type counterparts, CBFB-overexpressing T47D cells exhibited significantly downregulated PARP1, upregulated NFATC3 and NOS2 (Figure 4(d)), increased migration and invasion abilities (Figure 4(e)), and markedly increased Runx2, vimentin, Snail, CD44, and OPN expressions (Figure 4(f)). Moreover, we examined the probable effect of downregulated or overexpressed CBFB on the self-renewal ability of human breast cancers cells, using MDA-MB-436 and T47D cells tumorspheres, and the results demonstrated that CBFB expression caused significant tumorspheres formation compared with their control counterparts (Figure 4(g)). Interestingly, silencing or overexpressing CBFB affects the expression of several molecular factors. To explore potential possible mechanisms, we perform CO-IP and check the interaction between factors affected after altering CBFB expression as shown in Figure 4(h).

### 3.5. CBFB Silencing Significantly Suppresses Metastasis in the MDA-MB-436-Derived Tumor Xenograft Model, In Vivo

**Figure 5:**
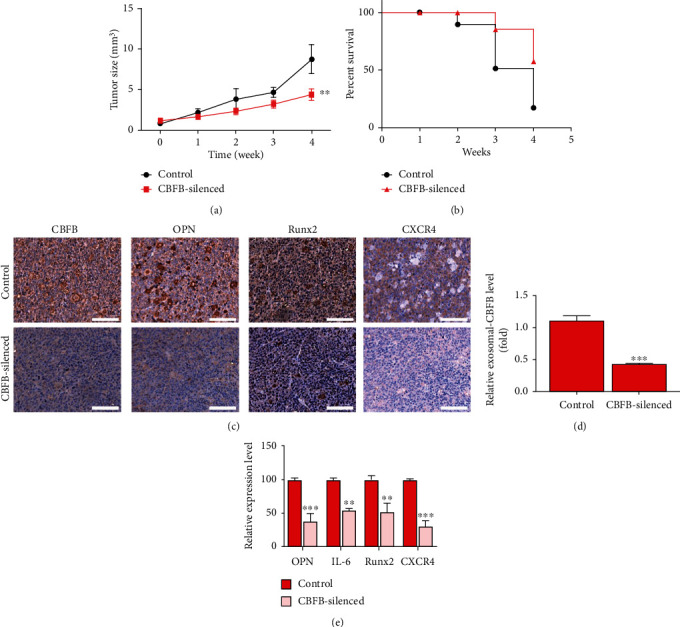
Downregulation of CBFB led to decreased bone metastasis in vivo. (a) Graph of tumor size over time indicating that the tumor burden of the CBFB-knockdown group was significantly lower than that of their control counterparts, ^∗∗^*p* < 0.01. (b) Kaplan–Meier survival curve indicating that CBFB-knockdown mice exhibited a significantly higher percentage survival than their control counterparts. (c) IHC images of the differential expression of CBFB, OPN Runx2, and CXCR4 protein levels of the control and CBFB-knockdown mice. (d) CBFB level in the exosomes isolated from the pooled serum of CBFB-knockdown mice was significantly lower than that of their control counterparts. (e) qPCR examination of bone metastasis-associated circulating markers in the serum of both control and CBFB-knockdown mice. CBFB-knockdown tumor samples exhibited a significantly lower level of CXCR4, OPN, Runx2, and IL-6 than did their control counterpart. ^∗∗^*p* < 0.01, ^∗∗∗^*p* < 0.001.

**Figure 6:**
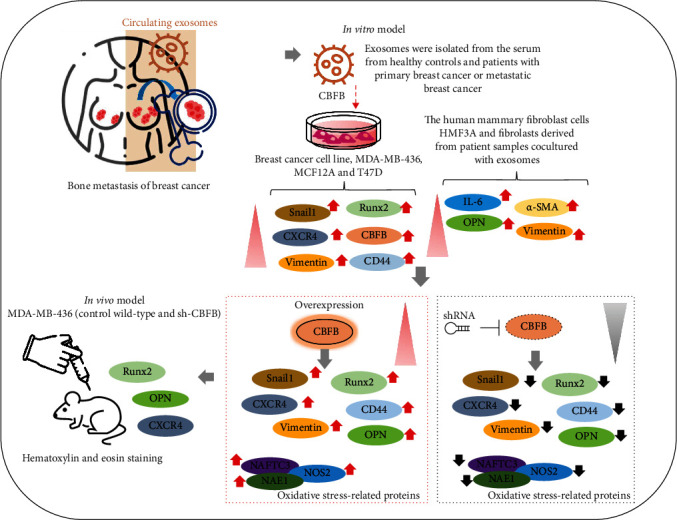
Metastatic breast cancer cells secrete exosomes rich in CBFB and facilitate the transformation of cancer-associated fibroblast-like phenotype cells (CAF-like phenotype cells). CAF-like cells, and exosomal CBFB in turn promote epithelial-to-mesenchymal (EMT) transition of breast cancer cells, prompting increased bone metastasis incidence.

For *in vivo* validation of data obtained from *in vitro* experiments, we used MDA-MB-436-derived xenograft mouse models generated by orthotopic injection of wild-type or CBFB-knockdown MDA-MB-436 cells into the mammary fat pads of NOD-SCID female mice. The size of tumors formed in mice injected with CBFB-knockdown cells was significantly smaller at the indicated time points, compared with that of the control group, with a 3.16-fold difference in tumor size by week 4 (*p* < 0.01) (Figure 5(a)). In addition, mice bearing CBFB-knockdown tumor cells had a significantly higher survival rate (week 4: control, 10% vs. CBFB-knockdown, 60%) (Figure 5(b)). Experiments using tumor samples derived from the tumor xenograft mouse models demonstrated that the expression of CBFB, OPN, Runx2, and CXCR4 proteins was significantly suppressed in CBFB-knockdown mice, compared with the control mice (Figure 5(c)). Compared with the control group, the CBFB-knockdown mice revealed significantly lower serum levels of exosomal CBFB (2.75-fold, *p* < 0.001) (Figure 5(d)) and significantly lower circulating serum levels of OPN (2.86-fold, *p* < 0.001), IL-6 (2.04-fold, *p* < 0.01), Runx2 (2.08-fold, *p* < 0.01), and CXCR4 (3.85-fold, *p* < 0.001) (Figure 5(e)). These data suggest a critical role for CBFB in the tumorigenesis and bone metastasis of breast cancer, with associated modulation of markers of bone metastasis, including Snail, CD44, vimentin, OPN, Runx2, and IL-6, as summarized in Figure 6.

## 4. Discussion

Breast cancers commonly metastasize to the bone. Despite substantial therapeutic advancements, treatment options for patients with breast-to-bone metastasis remain limited, and the prognosis remains poor. To develop more effective treatment strategies against breast-to-bone metastasis, a more thorough understanding of the underlying mechanisms is essential. In this study, we demonstrated for the first time, to the best of our knowledge, that elevated levels of the oncogene CBFB are associated with poor survival rates in patients with TNBC.

Increased CBFB activity has been associated with increased breast cancer invasiveness [15, 20]. Consistent with such findings, our results revealed significantly higher CBFB expression in the metastatic breast cancer cell lines MDA-MB-157 and MDA-MB-436 than in the less metastatic MCF12A and T47D cells; moreover, increased CBFB levels positively correlated with poor prognosis in patients with TNBC (Figure 1). CBFB mRNA levels were significantly higher in the sera of patients with bone metastatic breast cancer than in the sera of those with lung or brain metastasis or no metastasis. These findings agree with those of Ran et al., who indicated that CBFB plays an essential role in the maintenance of the mesenchymal phenotype of breast cancer cells and contributes to the formation of bone metastases [21].

Oxidative stress mechanisms activate cell signaling pathways, including tumor cell proliferation, tumor cell migration, and tumor cell proangiogenic factors, and play a key role in apoptosis. They can also affect cancer progression and metastasis. Increased ROS and the resulting high oxidative stress are key features of malignant tumors. Breast cancer cells are susceptible to oxidative damage and exhibit high levels of oxidative stress, including protein damage, DNA damage, and lipid peroxidation. Breast cancer is a common type of cancer and the main cause of cancer-related deaths in women. In the past few decades, targeted therapies for breast cancer have developed rapidly. Among these, MLN4924 is a first-class NEDD8 activating enzyme (NAE) inhibitor. In many studies, it has been revealed to exert antitumor activity by inactivating cullin-RING ligase and causing its substrate to accumulate to induce apoptosis. In this study, we observed that the abnormal performance of CBFB may affect the regulation of NAE1.

We also demonstrated that the CBFB-rich serum exosomes from patients with bone metastasis facilitated the acquisition of metastatic phenotypes by breast cancer cell lines that were hitherto less metastatic or nonmetastatic (Figure 2); these enhanced migration and invasion capabilities were associated with an increased expression of EMT markers, such as Snail, vimentin, and CD44, as well as bone metastasis markers OPN and Runx2. These findings are consistent with current knowledge that EMT alters cancer cell behavior with upregulated expression of mesenchymal proteins, such as Snail, Slug, vimentin, and Twist, and enhances cell migration and invasion [3, 8]. CD44 activates cell signaling pathways that drive cell proliferation, cell survival, and enhanced cell motility. Increased CD44 expression on circulating cancer cells enhances the “efficiency of post-intravasation events and distant metastasis in vivo, consistent with its association with increased distant recurrence and reduced disease-free survival in patients” [6, 7]. Both OPN and Runx2 are involved in the shaping of the TME for bone metastasis [22]. Notably, we observed elevated CBFB levels in the serum exosomes isolated from patients with bone metastasis compared with their nonmetastatic counterparts (Figure 2). Thus, serum exosomes from patients with metastatic disease may contain oncogenic and metastatic signaling molecules that transform nonmetastatic breast cancer cells into metastatic lineages. This would be consistent with the prevalent understanding that the extracellular vesicles, including exosomes, secreted by cancer cells are different from other forms of cell–cell communication because of not only their capacity for bulk transportation and organ tropism but also their critical roles in the initiation and dissemination of metastatic breast cancer [23].

When we cocultured fibroblasts with exosomes, the cells exhibited significantly increased expression of markers of CAFs such as vimentin and *α*-SMA; these cells exhibited increased secretion of OPN and IL-6 into the culture medium compared with the singly cultured normal fibroblasts. This is in line with reports that CAF-secreted IL-6 induces OPN, which itself is significantly upregulated in head and neck carcinomas and correlated with poor prognosis. Moreover, activated IL-6-OPN signaling during fibroblast–cancer cell interaction enhances cancer cell growth, migration, and invasion [24, 25]. Notably, increased serum OPN levels are predictive of cancer, especially metastatic cancer [26] and associated with a higher predisposition to bone metastasis [27, 28]. Therefore, our findings broaden the contemporary understanding of bone metastasis, especially that initiated or maintained by cancer-derived exosomes, as well as demonstrate the contribution of these cancer-derived exosomes in the acquisition of the CAF-like phenotype by normal stromal fibroblasts (Figure 3).

We also observed that CBFB knockdown significantly reduced both the migration and invasion abilities in the highly metastatic MDA-MB-436 cells, whereas CBFB overexpression increased these abilities in the less metastatic T47D cells (Figure 4). Consistent with these findings, a study reported that CBFB and Runx2 play essential roles in the expression of genes that mediate the ability of metastatic breast cancer cells to control osteoclast and osteoblast activities [20]. Oxidative stress regulates cellular function, reactive oxygen species (ROS), and antioxidant defenses that regulate intracellular signaling to prevent oxidative damage in a variety of pathological conditions. The RUNX2 DNA-binding transcription factor is activated by a glucose-mediated intracellular pathway, plays an important role in endothelial cell (EC) function and angiogenesis, and is a target of oxidative stress. Furthermore, Runx2 is a positive regulator of chondrocyte maturation and is involved in vascular invasion of cartilage. Core-binding factor beta (Cbfb) is a cotranscription factor that forms a heterodimer with Runx protein. The functionality of Runx1 and Runx2 requires Cbfb. Thus, the Runx2/Cbfb heterodimer plays an important role in skeletal development. In our results, the relevance of this regulation is preliminarily demonstrated (Figure 4). Moreover, STUB1 binds to RUNX1 and induces its ubiquitination and degradation mainly in the nucleus. STUB1 (also known as CHIP) is an E3 ubiquitin ligase that promotes the degradation of Runx1 and Runx2. Therefore, we also determined STUB1 (E3 ligase) associated with CBFB which overexpress after silencing CBFB. It might decline in expression of many factors and cause proteolysis (Supplementary Figure S2). Notably, we observed that silencing CBFB in MDA-MB-436 cells resulted in the reduced expression of EMT regulator Snail, metastasis/cancer stem cell marker CD44, and bone metastasis–associated markers OPN and Runx2. These findings hint at the essential roles of CBFB in maintaining the TNBC cell mesenchymal phenotype and may explain why mesenchymal-to-epithelial transition and reorganization of cells into epithelial breast cell-reminiscent acini-like structures are observed upon CBFB depletion [21]. Moreover, we observed lower levels of CBFB in the exosomes released by CBFB-knockdown MDA-MB-436 and MDA-MB-157 cells, whereas the opposite was true for the CBFB-overexpressing T47D and MCF12A cells, indicating that the total cellular CBFB mRNA level dictates the amount of CBFB packaged into the exosomes. Currently, most anticancer therapeutics increase ROS levels and alter the redox homeostasis in cancer cells, thus highlighting the importance of targeting oxidative stress in the treatment of patients with cancer. Our data indicated that CBFB knockdown significantly suppressed NAE1, NAFTC3, and NOS2 (Figure 4). In other words, similar to most chemotherapeutics, targeting CBFB elicits an anticancer effect by inducing ROS-mediated cell injury and oxidative stress in TNBC cells. Accumulating evidence indicates the critical role of altered cellular stress and impaired oxidative status in response to chemotherapy, especially the increasingly acclaimed benefits of oxidative stress modulators as a therapeutic strategy for patients with aggressive breast cancer [29–32].

Studies have revealed that CXCR4 is essential for the in vivo proliferation or survival of breast cancer cells, and its suppression may improve treatment response in patients with primary or metastatic breast cancer [33, 34]. Consistent with these findings, we demonstrated that CBFB silencing significantly reduced tumor burden; incidence of bone metastasis; and levels of CXCR4, OPN, Runx2, and IL-6 proteins and increased the survival rate in MDA-MB-436-injected tumor xenograft mouse models compared with their control counterparts (Figure 5). An increased level of OPN in tumor cells and tissues predicts a higher incidence of bone metastasis [35]. Similarly, increased expression of Runx2, another key bone metastasis marker in breast cancer, has been associated with increased TGF-*β* signaling and enhanced bone colonization of breast cancer cells [36–38]. Accordingly, decreased OPN and Runx2 expression following CBFB-knockdown suggests a potential role for CBFB in the treatment of bone metastasis in patients with breast cancer. Mitochondria are highly dynamic organelles that are remodeled to meet the metabolic demands of the cell. In the tumor microenvironment, due to rapid metabolism and hypoxia, cancer cells are often in a state of chronic inflammation, and at the same time, the concentration of reactive oxygen species (ROS) is higher than that of normal cells. Under normal conditions, appropriate reactive oxygen species can participate in physiological functions such as regulation of growth and development, metabolism, and elimination of microorganisms in cells; however, the increase in the concentration of reactive oxygen species also stimulates the transmission of many growth signals, resulting in cancer cell resistance and inhibition. The immune response eventually leads to tumor progression and metastasis. circ-CBFB knockdown attenuated mitochondrial dynamics perturbation and cellular damage. Previous studies have found that it works by triggering perturbations in mitochondrial dynamics. circ-CBFB acts as a ceRNA to play a regulatory role during cell injury, which may provide a potential therapeutic target [39].

Finally, our findings suggest that these molecules might be used collectively as a prognostic signature for bone metastasis and survival in clinical settings. However, this hypothesis should be validated in large-scale trials.

## 5. Conclusion

As shown in our pictorial abstract in Figure 6, we demonstrated the essential role of CBFB in the promotion of bone metastasis in breast cancer cells, as evidenced by its high expression in the tumor-derived or serum exosomes of patients with bone metastatic breast cancer. Breast cancer cells have high levels of oxidative stress, leading to oxidative damage, including protein damage, DNA damage, and lipid peroxidation. In addition, some breast cancer risk factors may change the level of endogenous oxidative stress. In this study, CBFB knockdown decreased tumor burden, bone metastasis, and markers of bone metastasis, including CXCR4, Snail, CD44, OPN, Runx2, and IL-6. CBFB may thus serve as a novel therapeutic target for bone metastasis in patients with breast cancer.

## Data Availability

The datasets used and analyzed in the current study are publicly accessible as indicated in the manuscript.

## Supplementary Materials

Supplementary Materials Supplementary Table S1: primers used in the present study. Supplementary Table S2: antibodies used in the present study. Supplementary Figure S1: CBFB mRNA expression across breast cancer cell lines (50) divided into primary and metastatic-derived breast cancer. The expression level data were downloaded from the cBioPortal website. CBFB levels were not different between the groups. NS: not significant. Supplementary Figure S2: STUB1 (E3 ligase) associated with CBFB which overexpress after silencing CBFB. Western blot raw data: Supplementary Figure S3: full-sized blots of Figure 2(a). Supplementary Figure S4: full-sized blots of Figure 2(d). Supplementary Figure S5: full-sized blots of Figure 3(c). Supplementary Figure S6: full-sized blots of Figure 4(a). Supplementary Figure S7: full-size blots of Figure 4(c). Supplementary Figure S8: full-sized blots of Figure 4(d). Supplementary Figure S9: full-size blots of Figure 4(f). Supplementary Figure S10: full-sized blots of Figure 4(h).
