# Supplementary material for: Cancer-Associated Exosomal CBFB Facilitates the Aggressive Phenotype, Evasion of Oxidative Stress, and Preferential Predisposition to Bone Prometastatic Factor of Breast Cancer Progression
Source: Dis Markers. 2022 Jul 19;2022:8446629. doi: 10.1155/2022/8446629 (PMC9325341; doi:10.1155/2022/8446629)
Supplement: Supplementary Materials — Supplementary Table S1: primers used in the present study. Supplementary Table S2: antibodies used in the present study. Supplementary Figure S1: CBFB mRNA expression across breast cancer cell lines (50) divided into primary and metastatic-derived breast cancer. The expression level data were downloaded from the cBioPortal website. CBFB levels were not different between the groups. NS: not significant. Supplementary Figure S2: STUB1 (E3 ligase) associated with CBFB which overexpress after silencing CBFB. Western blot raw data: Supplementary Figure S3: full-sized blots of Figure 2(a). Supplementary Figure S4: full-sized blots of Figure 2(d). Supplementary Figure S5: full-sized blots of Figure 3(c). Supplementary Figure S6: full-sized blots of Figure 4(a). Supplementary Figure S7: full-size blots of Figure 4(c). Supplementary Figure S8: full-sized blots of Figure 4(d). Supplementary Figure S9: full-size blots of Figure 4(f). Supplementary Figure S10: full-sized blots of Figure 4(h). [file 8446629.f1.docx]

**SUPPLEMENTARY INFORMATION**

**Cancer-associated exosomal CBFB facilitate the aggressive phenotype, evasion of oxidative stress, and preferential predisposition to bone prometastatic factor of breast cancer progression**

Chia-Hung Hsu^1,2,3^, Hon-Ping Ma^1,2,3^, Jiann Ruey Ong^1,2,3^, Ming-Shou Hsieh^4^, Vijesh Kumar Yadav^4^, Chi-Tai Yeh^4,5^, Tsu-Yi Chao^6^, Wei-Hwa Lee^7^, Wen-Chien Huang^8,9^, Kuang-Tai Kuo^4^, Iat-Hang Fong^4^, Chih-Cheng Lin^10^, and Chih-Ming Su^11,12^*

^1^ Department of Emergency Medicine, Shuang-Ho Hospital, Taipei Medical University, New Taipei City, Taiwan,

^2^ Graduate Institute of Injury Prevention and Control, College of Public Health, Taipei Medical University, Taipei City, Taiwan.

^3^ Department of Emergency Medicine, School of Medicine, Taipei Medical University, Taipei, Taiwan.

^4^ Department of Medical Research & Education, Taipei Medical University - Shuang Ho Hospital, New Taipei City 235, Taiwan.

^5^ Department of Medical Laboratory Science and Biotechnology, Yuanpei University of Medical Technology, Hsinchu City 30015, Taiwan.

^6^ Graduate Institute of Clinical Medicine, College of Medicine, Taipei Medical University, Taipei 110, Taiwan.

^7^ Department of Pathology, Taipei Medical University-Shuang Ho Hospital, New Taipei City, Taiwan.

^8^ Department of Medicine, MacKay Medical College, Taipei 110, Taiwan, ROC.

^9^ Division of Thoracic Surgery, Department of Surgery, MacKay Memorial Hospital, Taipei 110, Taiwan, ROC.

^10^ Department of Medical Laboratory Science and Biotechnology, Yuanpei University of Medical Technology, Hsinchu City 30015, Taiwan, ROC.

^11^ Division of General Surgery, Department of Surgery, School of Medicine, College of Medicine, Taipei Medical University, Taipei City, Taiwan.

^12^ Division of General Surgery, Department of Surgery, Department of Surgery, Taipei Medical University Shuang Ho Hospital, New Taipei City, Taiwan.

***** Corresponding Author:

Dr. Chih-Ming Su, MD., PhD. Division of General Surgery, Department of Surgery, School of Medicine, College of Medicine, Taipei Medical University, Taipei City, Taiwan. Phone: 886-2-2490088 ext. 8888, FAX: 886-2-2248-0900. E-mail: [su08261@tmu.edu.tw](mailto:su08261@tmu.edu.tw)

**Supplementary Table S1.** Primers used in the present study.

| **Gene** | **Forward** | **Reverse** |
| --- | --- | --- |
| Snail | TCGGAAGCCTAACTACAGCGA | AGATGAGCATTGGCAGCGAG |
| CXCR4 | ACTACACCGAGGAAATGGGCT | CCCACAATGCCAGTTAAGAAGA |
| OPN | CTGGTGCTCGTCCTCTACTAC | GGACACGAAGGTAAAGGTGAC |
| CBFB | AGAAGCAAGTTCGAGAACGAG | CCTGAAGCCCGTGTACTTAATCT |
| GAPDH | TGTGGGCATCAATGGATTTGG | ACACCATGTATTCCGGGTCAAT |
| circ-CBFB | CAGCGCTCAATCCTTTGGGA | GACCTGCCACATTGGTCAGTA |
| Runx2 | TGGTTACTGTCATGGCGGGTA | TCTCAGATCGTTGAACCTTGCTA |

**Supplementary Table S2.** Antibodies used in the present study.

| No. | Target | Dilution | Source | |
| --- | --- | --- | --- | --- |
| 1 | OPN | 1:500 | ab69498 | abcam |
| 2 | CD44 | 1:1000 | #5640 | Cell Signaling |
| 3 | CXCR4 | 1:1000 | #97680 | Cell Signaling |
| 4 | Runx2 | 1:500 | #12556 | Cell Signaling |
| 5 | Snail | 1:1000 | #3895 | Cell Signaling |
| 6 | Vimentin | 1:1000 | #5741 | Cell Signaling |
| 7 | CBFB | 1:500 | PA1-317 | ThermoFisher |
| 8 | CD9 | 1:500 | 5G6 | Novus Biologicals |
| 9 | CD63 | 1:500 | H5C6 | Novus Biologicals |
| 10 | GAPDH | 1:10000 | #5174 | Cell Signaling |
| 11 | Tubulin | 1:10000 | 11224-1-AP | PROTEINTECH |

Skin mets

Brain mets

**Supplementary Figure S1.** CBFB mRNA expression across breast cancer cell lines (50) divided into primary and metastatic-derived breast cancer. The expression level data were downloaded from the cBioprtal website. CBFB levels were not different between the groups. NS (not significant).

**Supplementary Figure S2.** STUB1 (E3 ligase) associated with CBFB which overexpress after silencing CBFB.

**
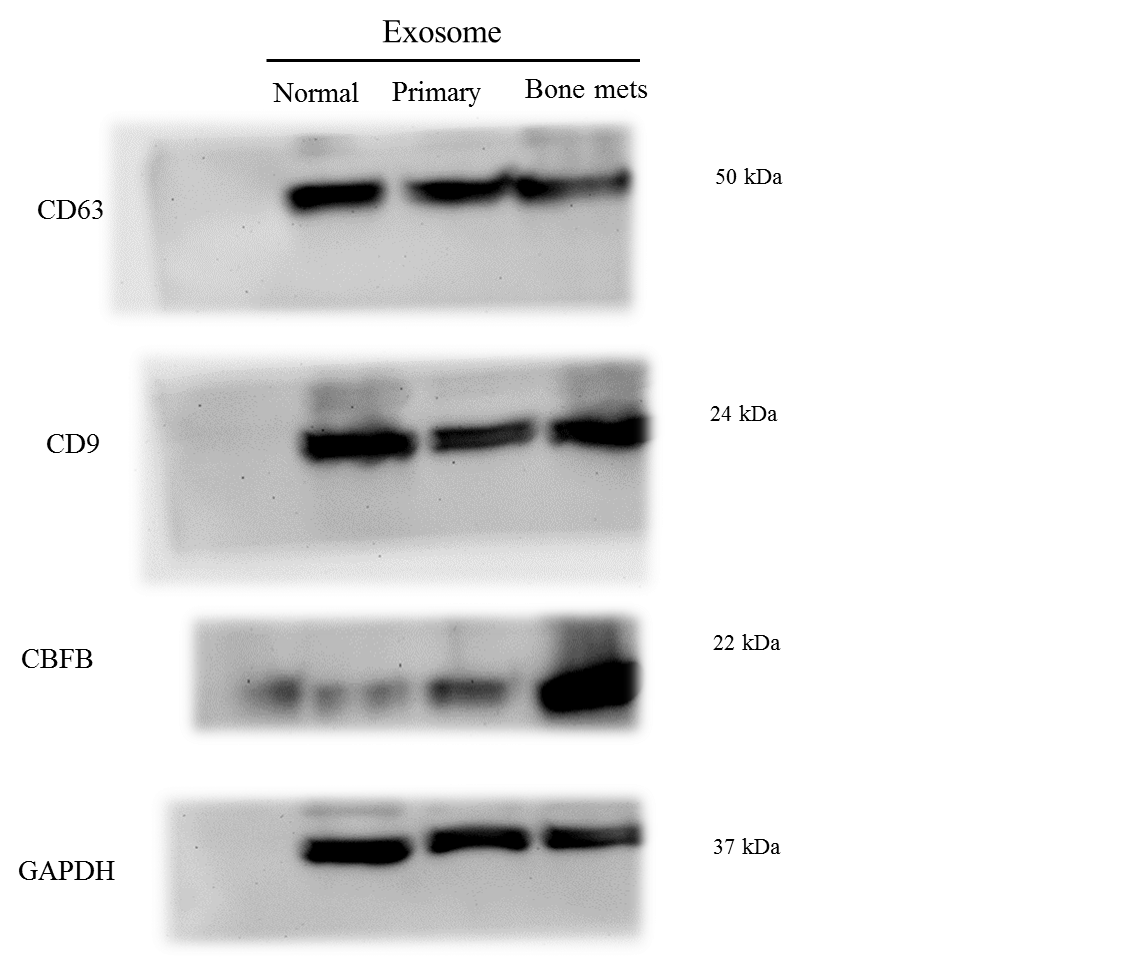
**

**Supplementary Figure S3.** Full-size blots of Figure 2A

**
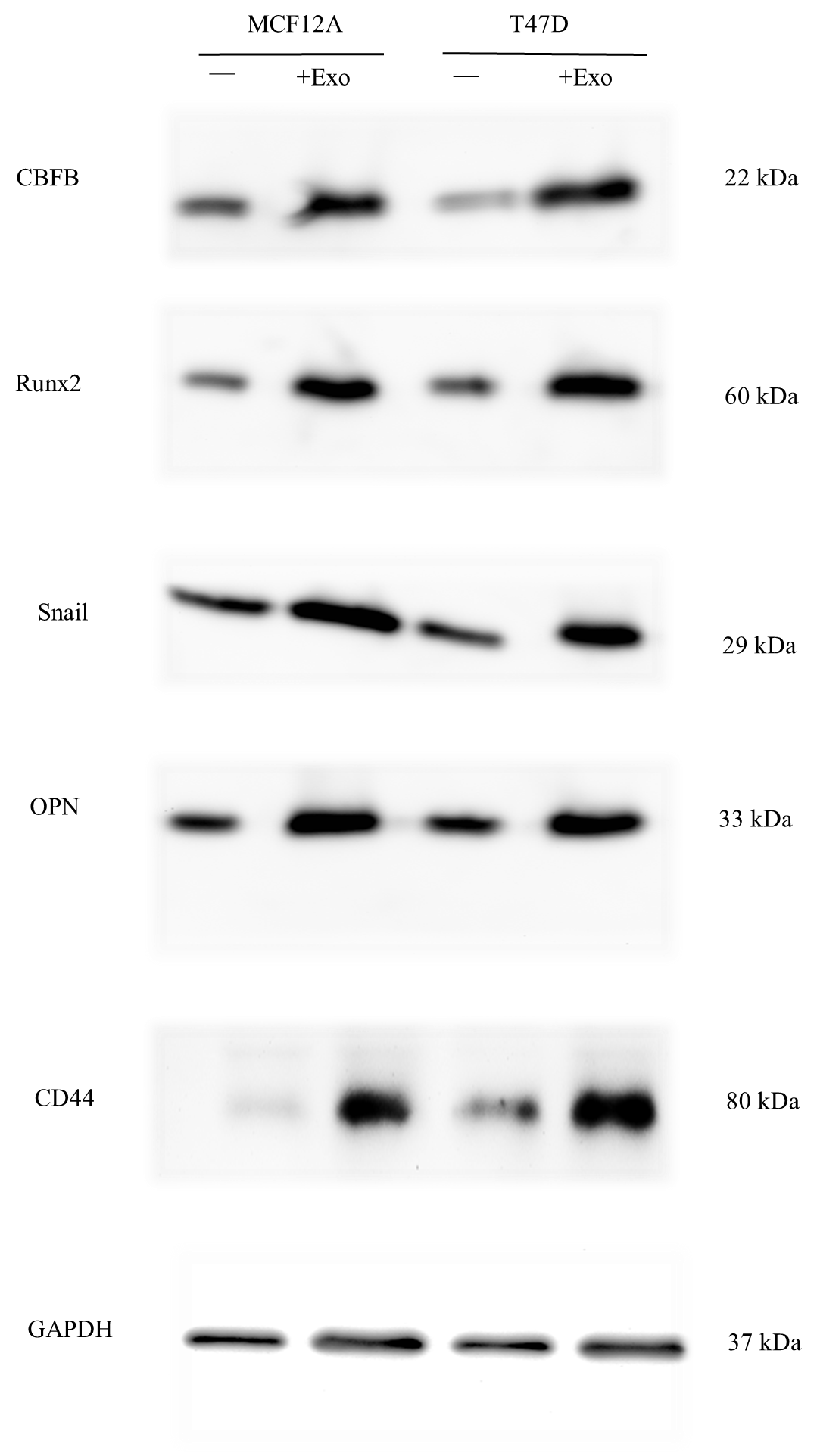
**

**Supplementary Figure S4.** Full-size blots of Figure 2D


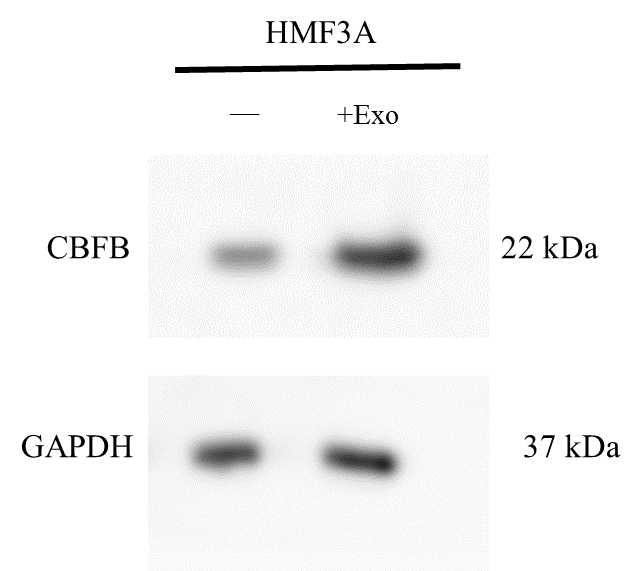


**Supplementary Figure S5.** Full-size blots of Figure 3C


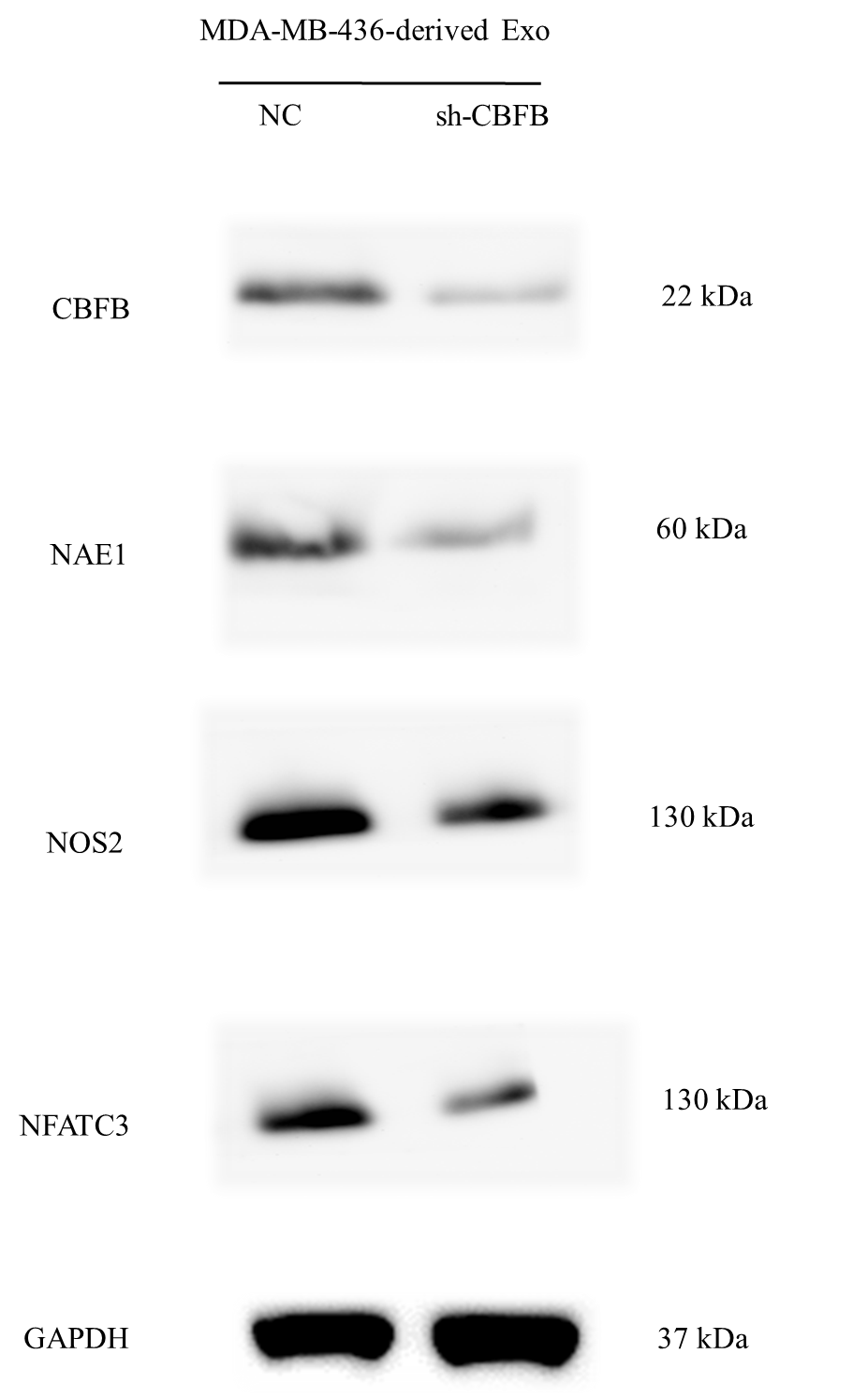


**Supplementary Figure S6.** Full-size blots of Figure 4A


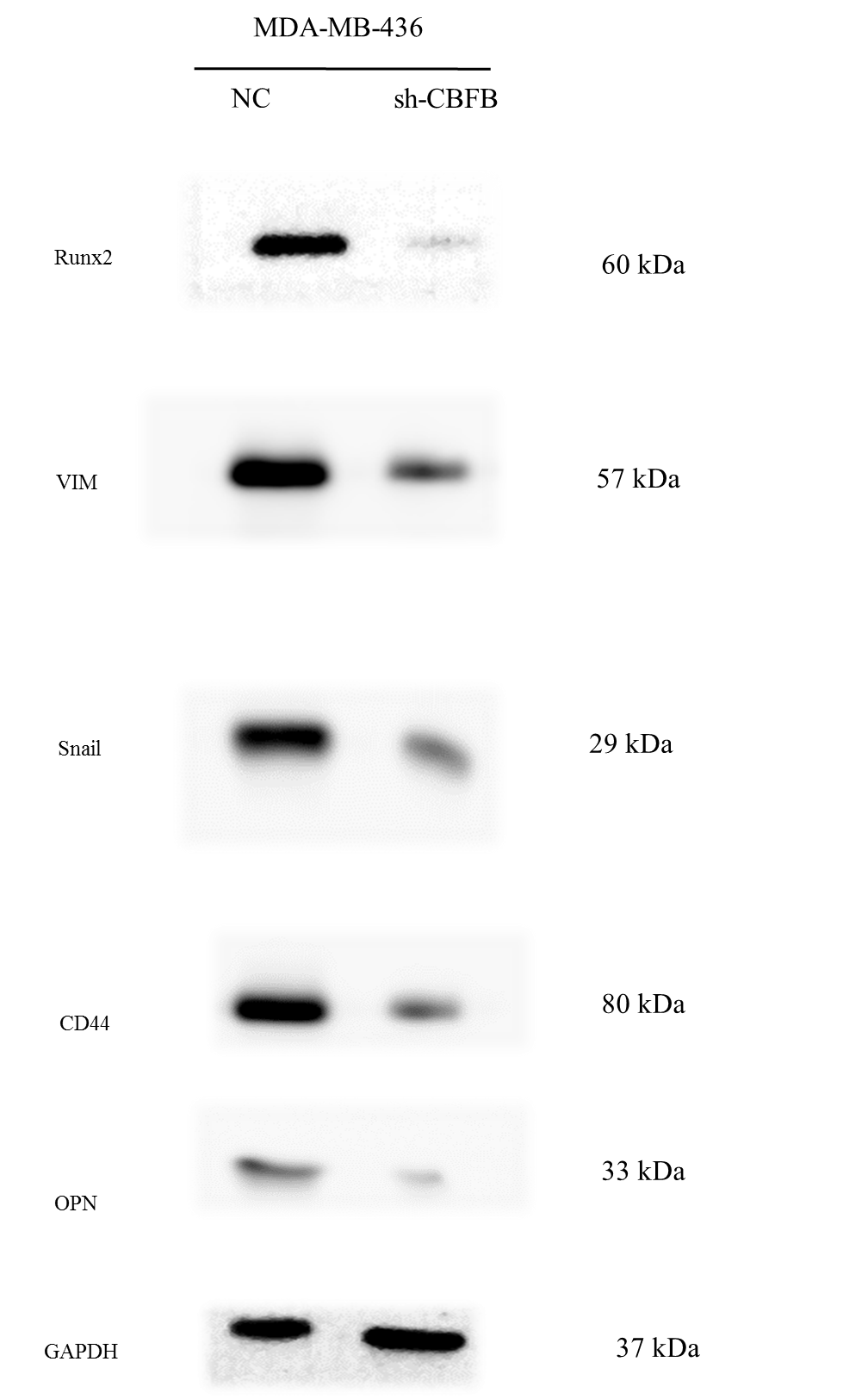


**Supplementary Figure S7.** Full-size blots of Figure 4C


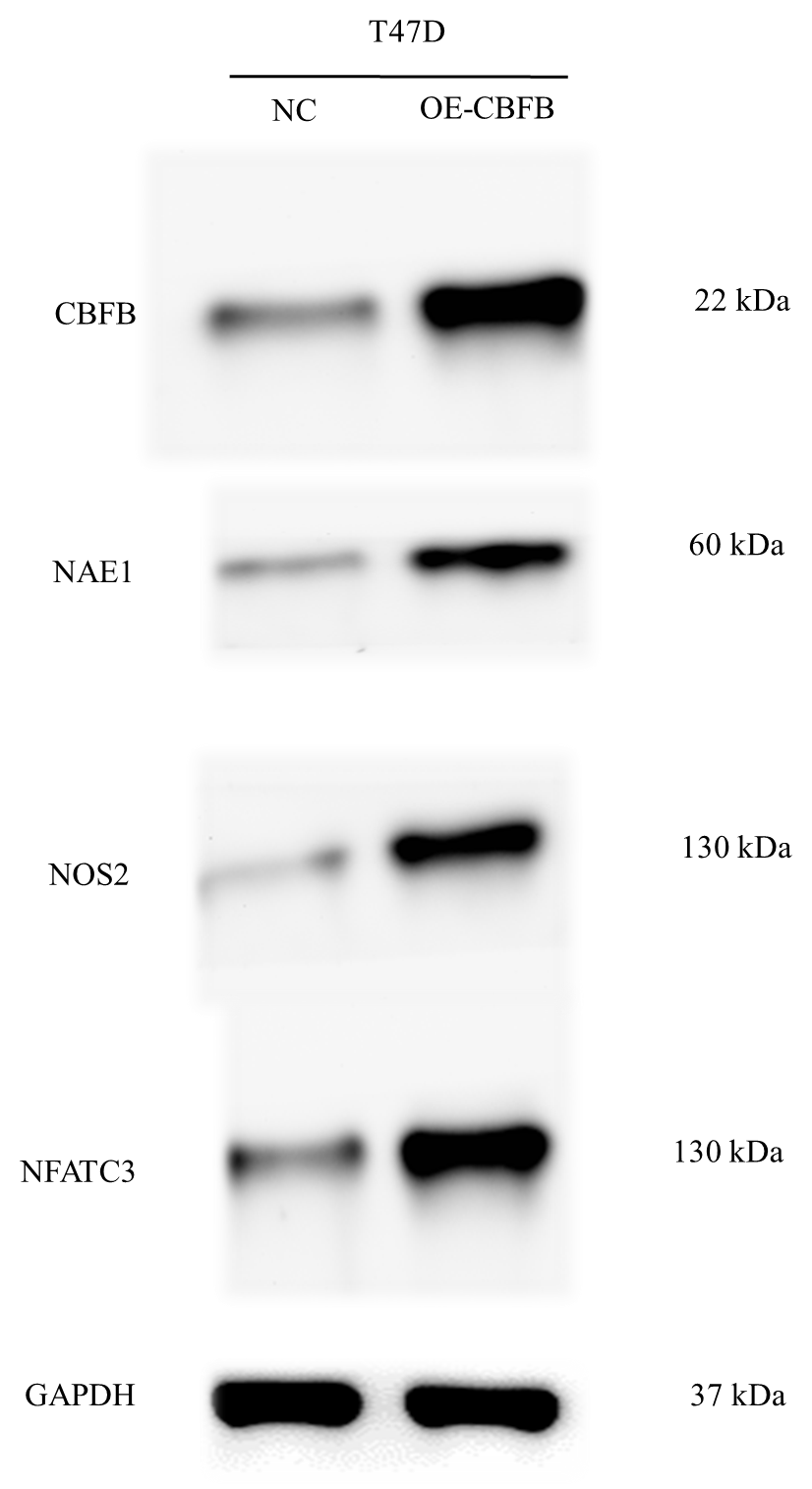


**Supplementary Figure S8.** Full-size blots of Figure 4D


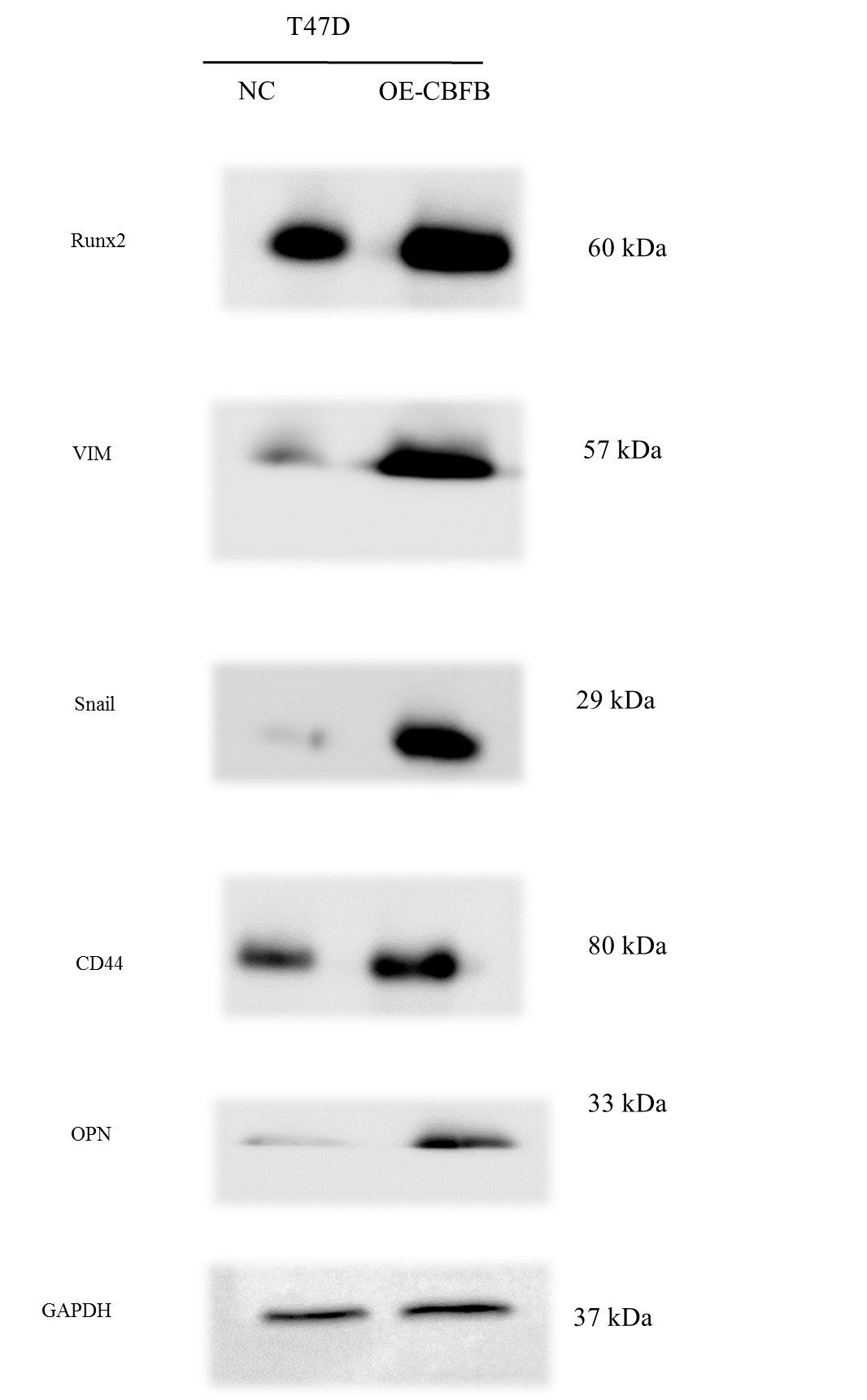


**Supplementary Figure S9.** Full-size blots of Figure 4F


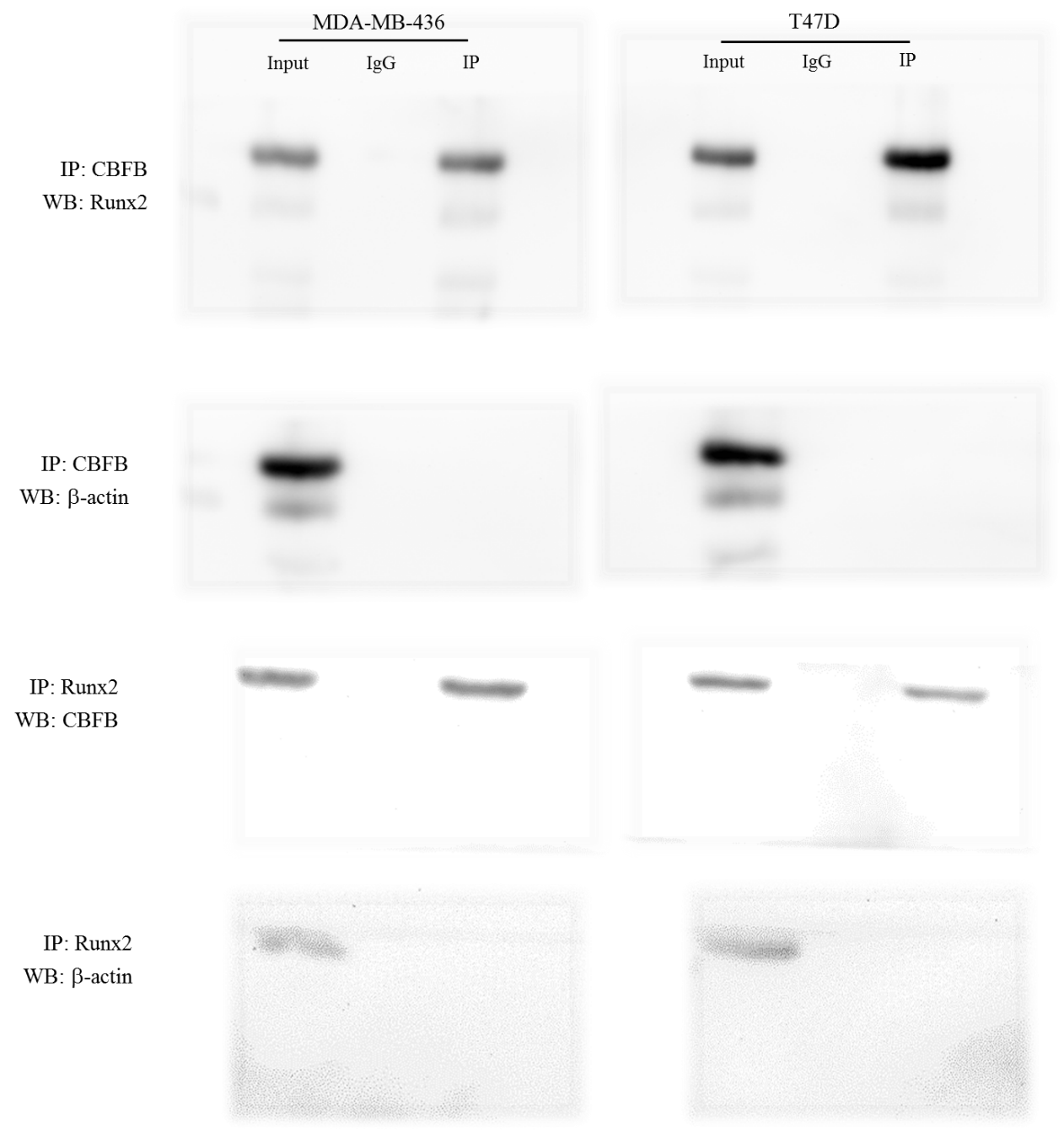


**Supplementary Figure S10.** Full-size blots of Figure 4H
